# Gyejigachulbu-Tang Relieves Oxaliplatin-Induced Neuropathic Cold and Mechanical Hypersensitivity in Rats via the Suppression of Spinal Glial Activation

**DOI:** 10.1155/2014/436482

**Published:** 2014-11-17

**Authors:** Byung-Soo Ahn, Seong-Kyu Kim, Ha Neul Kim, Ji-Hye Lee, Ji-Hwan Lee, Deog Sang Hwang, Hyunsu Bae, Byung-Il Min, Sun Kwang Kim

**Affiliations:** ^1^Department of East-West Medicine, Graduate School, Kyung Hee University, Seoul 130-701, Republic of Korea; ^2^Department of Physiology, College of Korean Medicine, Kyung Hee University, Seoul 130-701, Republic of Korea; ^3^Department of Microbiology, Pusan National University, Busan 609-735, Republic of Korea; ^4^Department of Gynecology, College of Korean Medicine, Kyung Hee University, Seoul 130-701, Republic of Korea; ^5^Department of Physiology, College of Medicine, Kyung Hee University, Seoul 130-701, Republic of Korea

## Abstract

Activation of spinal glial cells plays a crucial role in the pathogenesis of neuropathic pain. An administration of oxaliplatin, an important anticancer drug, often induces acute neuropathic cold hypersensitivity and/or mechanical hypersensitivity in patients. Gyejigachulbu-tang (GBT), a herbal formula comprising* Cinnamomi Cortex, Paeoniae Radix, Atractylodis Lanceae Rhizoma, Zizyphi Fructus, Glycyrrhizae Radix, Zingiberis Rhizoma, *and* Aconiti Tuber*, has been used in East Asia to treat various pain symptoms, especially in cold patients. This study investigated whether and how GBT alleviates oxaliplatin-induced cold and mechanical hypersensitivity in rats. The behavioral signs of cold and mechanical hypersensitivity were evaluated by a tail immersion test in cold water (4°C) and a von Frey hair test, respectively. The significant cold and mechanical hypersensitivity were observed 3 days after an oxaliplatin injection (6 mg/kg, i.p.). Daily oral administration of GBT (200, 400, and 600 mg/kg) for 5 days markedly attenuated cold and mechanical hypersensitivity. Immunoreactivities of glial fibrillary acidic protein (GFAP, astrocyte marker) and OX-42 (microglia marker) in the spinal dorsal horn were significantly increased by an oxaliplatin injection, which were restored by GBT administration. These results indicate that GBT relieves oxaliplatin-induced cold and mechanical hypersensitivity in rats possibly through the suppression of spinal glial activation.

## 1. Introduction

Oxaliplatin is a third-generation platinum-based chemotherapy drug that has gained importance in the treatment of advanced colorectal cancer [1, 2]. Unlike the other platinum compounds (e.g., cisplatin), oxaliplatin does not induce a nephrotoxicity but often induces a very acute painful neuropathy even from a single administration [3]. This acute neurotoxic side effect, the only major dose-limiting toxicity associated with oxaliplatin use [4], is characterized by the rapid onset of spontaneous severe pain and cold hypersensitivity in the hands, feet, perioral area, or throat [5–7]. Previous animal studies also have shown that a single injection of 6 mg/kg oxaliplatin reproduces the specific sensory neurotoxic profile, especially cold hypersensitivity, without hyperalgesia or allodynia to heat stimuli [8, 9]. However, the mechanism and treatment of these neuropathic symptoms have not been established yet [10], suggesting that novel therapeutic options are critically needed.

Two decades ago, glial cells in the spinal cord have been shown to contribute to peripheral neuropathic pain [11, 12]. Many lines of evidence have revealed that spinal astrocytes and microglia change their morphology and function following peripheral nerve damage. These activated glial cells release several cytokines and molecules that directly affect synaptic transmission and excitability in neighboring neurons, resulting in behavioral hypersensitivity (i.e., allodynia) [11–14]. Although a recent study [15] showed satellite glial cell activation in the dorsal root ganglia of oxaliplatin-treated mice, it is still unknown whether spinal glial activation plays a role in this oxaliplatin-induced neuropathic hypersensitivity.

In traditional herbal medicine in East Asia including China, Japan, and Korea, GBT that is composed of several “hot” medicinal herbs has long been used to treat several pain symptoms, especially in cold patients. Interestingly, Buja (Bushi in Japanese, a processed* Aconiti tuber*), one of the key components in this herbal formula, was recently known to inhibit spinal glial activation and neuropathic allodynia following peripheral nerve injury [16]. In the present study, we investigated whether GBT relieves oxaliplatin-induced cold and mechanical hypersensitivity and if so, whether such antiallodynic effects of GBT are related to the modulation of spinal glial activation.

## 2. Materials and Methods

### 2.1. Animals

Male Sprague-Dawley rats (160–210 g; Daehan Biolink, Chungbuk, Korea) were housed in cages (4 rats per cage) with water and food available* ad libitum*. The room was maintained with a 12 h light/dark cycle (a light cycle; 08:00–20:00, a dark cycle; 20:00–08:00) and kept at 23 ± 2°C. All animals were acclimated in their cages for 1 week prior to any experiments. The total number of rats used in this study was 77. All procedures involving animals were approved by the Institutional Animal Care and Use Committee of Kyung Hee University [KHUASP(SE)-13-047] and were conducted in accordance with the guidelines of the International Association for the Study of Pain [17].

### 2.2. Oxaliplatin Administration

To induce neuropathic cold allodynia in rats [18, 19], oxaliplatin (Sigma, USA), dissolved in a 5% glucose (Sigma, USA) solution at a concentration of 2 mg/mL, was intraperitoneally administered at 6 mg/kg. The same volume of 5% glucose solution was intraperitoneally injected in a vehicle control group.

### 2.3. Behavioral Tests

As previously described [20, 21], the behavioral signs of cold allodynia were determined by a tail immersion test in cold water. Briefly, each animal was lightly immobilized in a plastic holder and its tail was drooped for a proper application of cold water stimuli. The rats were adapted to the holder for 2 days before initiating behavioral tests. The tail was immersed in 4°C water, and then the tail withdrawal latency was measured with a cut-off time of 15 sec. The tail immersion test was repeated five times at 5 min intervals. When calculating the average latency, the cut-off time was assigned to the normal responses. The average latency was taken as a measure for the severity of cold allodynia; shorter tail withdrawal latency was interpreted as more severe allodynia. All behavioral tests were performed in blinded fashion.

For mechanical allodynia, we measured the withdrawal threshold of the tail in response to a series of von Frey hairs (log units: 3.61, 3.84, 4.08, 4.31, 4.56, 4.74, 4.93, and 5.18; equivalent in bending force to 0.4, 0.6, 1.0, 2.0, 4.0, 6.0, 8.0, and 15.0 g; Stoelting, USA). The 50% withdrawal threshold was determined using the up-down method [22]. Briefly, animals were placed into a plastic holder with the tail protruding on the table. Testing was initiated with a hair of which bending force was 2.0 g. The most sensitive spot of the tail was first determined by probing various areas with bending the hair for 1-2 s. If an abrupt tail withdrawal was observed (i.e., positive response), the filament with the next lower bending force was applied. When no response was observed, the next higher filament was applied. This testing procedure continued until a response to the fifth von Frey hair stimulation from the first change of response was measured. The responses were converted into a 50% threshold value using the following equation: 50% threshold (g) = *X*_*f*_ + *κδ*, where *X*_*f*_ is the value of the final von Frey filament (log units), *κ* is the correction factor (from calibration table), and *δ* is the mean difference of log units between stimuli [23]. A 15.0 g pressure was set as a cut-off value.

### 2.4. GBT Treatment and Experimental Groups

GBT (Keishikajutsubuto in Japanese), manufactured by Tsumura & Co., Japan (TJ-18, Lot: GBO 301), is a dried decoction of a mixture of seven medicinal herbs (Table 1). The quality of this herbal formula is strictly controlled by the manufacturer. In addition, a test sample was analyzed by three-dimensional high-performance liquid chromatography (HPLC) and the ingredients were checked (see Supplementary Figure  1 in the Supplementary Material available online at http://dx.doi.org/10.1155/2014/436482). This GBT was suspended and diluted in normal saline and the rats were orally injected by an oral gavage at the concentration of 200, 400, or 600 mg/kg/day for 5 days after an oxaliplatin injection. The concentration of GBT in this study is similar to that of the other herbal formula used in our previous study [24] and that of Buja in others' study [16].

The rats were randomly divided into the following six groups (*n* = 9 per group for cold and* n* = 3–5 per group for mechanical test): (1) NS/Vehicle (i.p. injection of 5% glucose solution + daily oral administration of normal saline), (2) GBT400/Vehicle (i.p. injection of 5% glucose solution + daily oral administration of 400 mg/kg GBT), (3) NS/Oxali (i.p. injection of oxaliplatin + daily oral administration of normal saline), (4) GBT200/Oxali (i.p. injection of oxaliplatin + daily oral administration of 200 mg/kg GBT), (5) GBT400/Oxali (i.p. injection of oxaliplatin + daily oral administration of 400 mg/kg GBT), and (6) GBT600/Oxali (oxaliplatin injection + daily oral administration of 600 mg/kg GBT). On day 0, a tail immersion test or von Frey hair test was performed and then oxaliplatin (6 mg/kg) or vehicle (5% glucose solution) was intraperitoneally injected, followed by an oral administration of GBT or saline. From day 1 to day 5, the behavioral test was daily performed 30 min after a GBT or saline administration.

### 2.5. Immunohistochemistry

The animals anesthetized with isoflurane were transcardially perfused with 50 mM phosphate-buffered saline (PBS) and fixed with a freshly prepared solution consisting of 4% paraformaldehyde in 100 mM phosphate buffer (pH 7.4). The sacral spinal cord segment at the S1 level was extracted and postfixed in the same fixative overnight and transferred into a 30% sucrose solution for cryoprotection. The tissues were sectioned at 30 *μ*m on a freezing microtome (Leica, Germany). The sections were incubated in PBS for 10 min, washed three times with PBS, and then incubated in 1% hydrogen peroxide (H_2_O_2_) for 30 min. Next, the sections were incubated overnight with mouse monoclonal anti-GFAP antibody (Chemicon, USA) at a dilution of 1 : 500 for visualization of astrocytes or with mouse anti-OX-42 antibody (Chemicon) at a dilution of 1 : 500 for visualization of microglia. The sections were then incubated for 1 h with anti-mouse secondary antibody (1 : 200; Vector Laboratories, USA) and the bound secondary antibody was amplified with a Vector Elite ABC Kit (Vector Laboratories). The sections were subsequently incubated with avidin-biotin-peroxidase complex (1 : 100; Vector Laboratories) for 1 h at room temperature. Immunoreactivity was visualized by incubating the sections in a solution consisting of 0.05% 3,3-diaminobenzidine (DAB) and 0.01% H_2_O_2_ in 50 mM Tris buffer (pH 7.6) for approximately 3 min. The sections were then mounted on gelatin-coated glass slides which were air-dried overnight at room temperature. Coverslips were mounted using Permount. To determine the mean number of reactive microglia and astrocytes, four to five sections per animal were randomly chosen for evaluation. Before beginning the image analysis, the light source was adjusted to the brightness that generates the best possible contrast between positive and negative stained microglia and astrocytes. The number of OX-42-positive microglia and GFAP-positive astrocytes was quantified in the spinal dorsal horn including laminae I–IV by a blinded observer [25].

### 2.6. Statistical Analysis

All the data are presented as mean ± S.E.M (standard error of the mean). Statistical analysis and graphic works were done with Prism 5.0 (Graph Pad Software, USA). One-way or two-way analysis of variance (ANOVA) followed by Dunnett's post hoc test was used for statistical analysis. In all cases, *P* < 0.05 was considered significant.

## 3. Results

### 3.1. Effects of GBT on Oxaliplatin-Induced Cold and Mechanical Hypersensitivity

**Figure 1:**
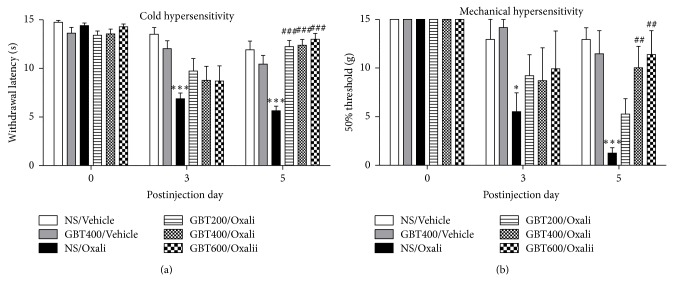
Effects of GBT on oxaliplatin-induced cold and mechanical hypersensitivity. (a) The average tail withdrawal latency in response to cold stimuli prior to (left) and 3 days (middle) and 5 days (right) after an oxaliplatin (6 mg/kg, i.p.) injection. (b) The mechanical threshold in response to von Frey hair stimuli prior to (left) and 3 days (middle) and 5 days (right) after an oxaliplatin (6 mg/kg, i.p.) injection. Data are presented as mean ± S.E.M.^*^ *P* < 0.05, ^***^ *P* < 0.001 versus NS/Vehicle; ^##^ *P* < 0.01, ^###^ *P* < 0.001 versus NS/Oxali by one-way ANOVA followed by Dunnett's post hoc test.

For all groups of rats, no abnormal clinical signs and no deterioration in general status and weight gain during the course of the experiment were observed. Similar to our previous results [19], the withdrawal latency in response to cold stimuli was nearly cut-off value (15 sec) prior to an oxaliplatin (6 mg/kg, i.p.) injection (Figure 1(a), day 0) and was significantly decreased from 3 days to at least 5 days following the injection (Figure 1(a), NS/Vehicle versus NS/Oxali, *P* < 0.001). The time course of mechanical threshold following an oxaliplatin injection also showed a similar change (Figure 1(b)).

The antiallodynic effects of GBT in oxaliplatin-injected rats are shown in Figure 1. GBT at different doses (200, 400, and 600 mg/kg) was orally administered every day after an oxaliplatin injection. Behavioral tests were performed 30 min after an oral administration of GBT on each experimental day. On the 3rd day after an oxaliplatin injection, there was no significant difference in cold and mechanical sensitivity between the normal saline-administered control group and GBT-administered groups, although slight increases in tail withdrawal latency and mechanical threshold were observed after GBT treatments. Finally, on the 5th day, all doses of GBT markedly attenuated the cold and mechanical hypersensitivity (*P* < 0.01, versus NS/Oxali), indicating a cumulative effect of GBT on cold allodynia. When a small subset of animals (*n* = 4 per groups) was tested before GBT administration, a similar cumulative effect of GBT on oxaliplatin-induced cold hypersensitivity was observed (Supplementary Figure  2). In vehicle-injected normal rats (i.e., no oxaliplatin injection), GBT treatment induced no change in cold and mechanical sensitivity (NS/Vehicle versus GBT400/Vehicle, *P* > 0.05).

### 3.2. Effects of GBT on Spinal Glial Activation

**Figure 2:**
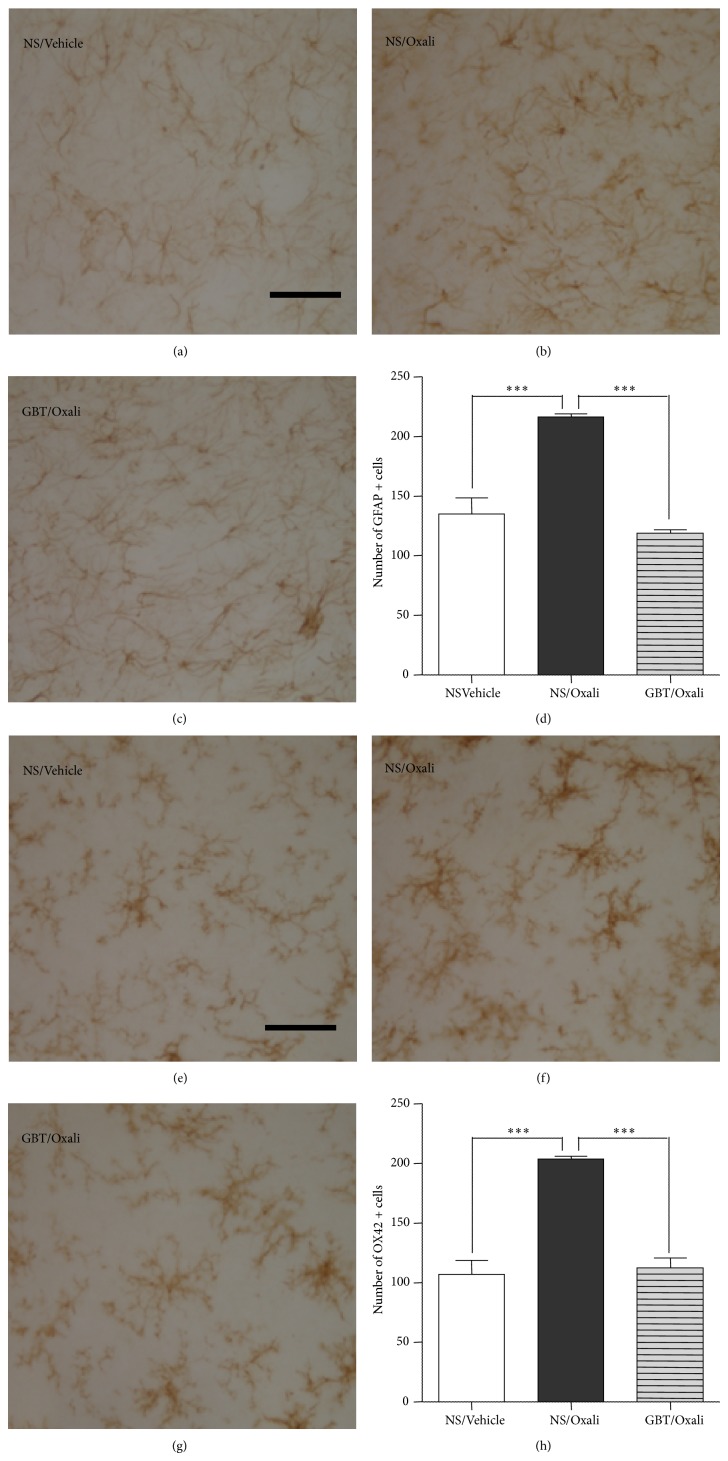
Suppressive effects of GBT on the activation of spinal glial cells. Immunohistochemical analysis of spinal dorsal horn laminae I–IV revealed a morphological activation (b) and an increase in the density (d) of GFAP-positive cells (astrocytes) by an oxaliplatin (6 mg/kg., i.p.) injection, compared to those in a vehicle-injected group (a, d). Such oxaliplatin-induced activation of spinal astrocytes was restored by GBT (400 mg/kg/day) treatments (c, d). Immunohistochemical analysis of spinal dorsal horn also showed a morphological activation (f) and an increase in the density (h) of OX42-positive cells (microglia) by an oxaliplatin injection, compared to those in a vehicle-injected group (e, h). Such microglial activation was restored by GBT treatments (g, h). Data are presented as mean ± S.E.M.^***^ *P* < 0.001 by one-way ANOVA followed by Dunnett's post hoc test. Scale bar, 50 *μ*m.

At the end of the behavioral experiments on the 5th day from an oxaliplatin injection, the spinal cord sections obtained from the animals were processed for immunohistochemical analyses of astrocytes and microglia activation (Figure 2). GFAP immunostaining results showed that spinal astrocytes in the NS/Oxali group were morphologically activated (i.e., hypertrophic with thick processes; Figure 2(b)), compared to those in the NS/Vehicle control group showing the resting state morphology with small soma and thin processes (Figure 2(a)). The oxaliplatin injection also significantly increased the density of GFAP-positive cells in the spinal cord dorsal horn laminae I–IV (*P* < 0.001, 216.3 ± 2.8 cells in the NS/Oxali group versus 135.0 ± 13.5 cells in the NS/Vehicle group; Figure 2(d)). Daily oral administration of GBT (400 mg/kg) restored the oxaliplatin-induced morphological activation of spinal astrocytes to the NS/Vehicle control level (Figure 2(c)) and significantly suppressed the increase in the number of spinal GFAP-positive cells (*P* < 0.001 versus NS/Oxali; Figure 2(d)). The GBT600/Oxali group showed similar suppressive effects on spinal astrocyte activation (117.2 ± 3.8 cells; *P* < 0.001 versus NS/Oxali), but GBT200/Oxali group did not (196.1 ± 14.6 cells; *P* > 0.05 versus NS/Oxali).

OX-42 immunostaining data showed that spinal microglia in the NS/Oxali group were morphologically activated (i.e., hypertrophic amoeboid shape with short processes; Figure 2(f)), compared to those in the NS/Vehicle control group showing the ramified resting state morphology with small soma and long processes (Figure 2(e)). The number of OX-42-positive cells in the spinal dorsal horn laminae I–IV was also significantly increased by an oxaliplatin injection (*P* < 0.001, 204.0 ± 2.2 cells in the NS/Oxali group versus 106.7 ± 11.9 cells in the NS/Vehicle group; Figure 2(h)). GBT (400 mg/kg) treatments restored the oxaliplatin-induced morphological activation of spinal microglia to the control level (Figure 2(g)) and significantly suppressed the increase in the number of spinal OX-42-positive cells (*P* < 0.001 versus NS/Oxali; Figure 2(h)). Both of GBT200/Oxali and GBT600/Oxali groups showed similar suppressive effects on spinal microglia activation (133.8 ± 10.3 cells and 133.5 ± 5.2 cells, resp.; *P* < 0.001 versus NS/Oxali). In vehicle-injected normal rats (i.e., no oxaliplatin injection), GBT treatment induced no change in spinal glial cells (NS/Vehicle versus GBT400/Vehicle: 135.0 ± 13.5 versus 142.0 ± 10.6 cells (GFAP-positive astrocytes); 106.7 ± 11.9 versus 84.7 ± 8.7 cells (OX-42-positive microglia), *P* > 0.05).

## 4. Discussion

Oxaliplatin has been used to treat patients with advanced and metastatic colorectal cancer and also has the effects against other cancers, including ovarian, breast, and lung cancers [1, 2]. However, acute neurotoxicity, characterized by cold hypersensitivity in the periphery, is developed even after a single oxaliplatin administration [8, 9]. Hence if oxaliplatin-induced peripheral neuropathy could be alleviated, the patients might overcome cancer without the cessation of chemotherapy.

Although the important role of spinal glial activation is now widely accepted in the pathogenesis of peripheral neuropathic pain, there have been contradictory results in the chemotherapy-induced peripheral neuropathy (CIPN). For example, Peters et al. [26] reported that paclitaxel induced an activation of both spinal astrocytes and microglia, but another group [27] showed that only spinal microglia, not astrocytes, were activated. Zheng et al. [28] further reported that the commonly used chemotherapy drugs including paclitaxel, vincristine, and oxaliplatin could not induce spinal microglia activation at all. A recent study [29] also demonstrated that oxaliplatin induced a transient activation of spinal microglia, but more prolonged activation of spinal astrocytes. Since those studies adopted different procedures (e.g., dose, frequency, and duration) for chemotherapy drug infusion, it might be reasonable to think that the contribution of spinal glial activation to CIPN is depending on the animal model. In this study, we found that both spinal astrocytes and microglia were significantly activated by an oxaliplatin injection (6 mg/kg, i.p.), which was correlated with the development of acute cold and mechanical hypersensitivity. These results suggest that spinal glial activation might play a role in oxaliplatin-induced acute neuropathic pain in rats.

Because interesting characteristic of the oxaliplatin-induced peripheral neuropathy is a strong cold hypersensitivity, we considered GBT as a potential alternative therapeutic option, based on the traditional medicine theory in East Asia as well as recent clinical evidence. GBT has its basis from the* Sang Han Lun* and was slightly modified in Japan during the Edo period [30]. As mentioned earlier, it is mainly composed of “hot-character” medicinal herbs, such as* Cinnamomi Cortex*,* Zingiberis Rhizoma, *and* Aconiti Tuber*. Thus, it has been traditionally used against “cold”-induced disorders including common cold, influenza, arthritis, neuralgia, and painful spasms. This traditional concept might be clinically important. Recent clinical studies indeed have shown that administration of GBT and Buja, a key component in GBT, significantly improves cold stimuli-evoked peripheral neuropathic pain in oxaliplatin-administered colorectal cancer patients or postherpetic neuralgia patients [31, 32]. Our previous study using the same rat model used in this study also showed that sweet bee venom, traditionally thought as having “hot-character,” potently relieves the oxaliplatin-induced cold allodynia [18]. Furthermore, Koizumi and his colleagues demonstrated that Buja strongly suppressed spinal glial activation following peripheral nerve damage, resulting in antiallodynia [16].

Inspired by those studies, we demonstrated here that GBT could suppress the spinal activation of astrocytes and microglia following an oxaliplatin injection and alleviate oxaliplatin-induced acute cold and mechanical hypersensitivity. Since the vehicle-injected normal control rats exhibited no change in tail withdrawal latency as well as in spinal glial cells after GBT treatment, our results indicate that GBT does not affect normal nociception but attenuates oxaliplatin-induced pathological hypersensitivity. However, it is still unknown which molecular mechanism is responsible for the suppressive effect of GBT or Buja on the spinal glial activation in oxaliplatin-injected or nerve-injured animals, respectively. In addition to this question, whether GBT can modulate peripheral transient receptor potential ankyrin 1 (TRPA1) or transient receptor potential melastatin 8 (TRPM8) channels, the well-known mediators for oxaliplatin-induced cold and mechanical hypersensitivity [33, 34], is to be investigated in our ongoing studies.

## 5. Conclusions

In conclusion, our findings in this study suggest that GBT potently alleviates oxaliplatin-induced acute cold and mechanical hypersensitivity via suppressing the activation of spinal astrocytes and microglia. Thus, GBT treatment can be an alternative therapeutic option in oxaliplatin-induced peripheral neuropathic pain.

## Supplementary Material

Supplementary Figure 1: Three-dimentional HPLC profile of GBT.
Supplementary Figure 2: Effects of GBT on oxaliplatin-induced cold hypersensitivity that was tested before GBT administration.

## Figures and Tables

**Table 1:** The components of GBT.

Medicinal herbs	Ratio
*Cinnamomi Cortex *	8
*Paeoniae Radix *	8
*Atractylodis Lanceae Rhizoma *	8
*Zizyphi Fructus *	8
*Glycyrrhizae Radix *	4
*Zingiberis Rhizoma *	2
*Aconiti Tuber *	1

## References

[1] Baker D. E. (2003). Oxaliplatin: a new drug for the treatment of metastatic carcinoma of the colon or rectum. *Reviews in Gastroenterological Disorders*.

[2] Screnci D., McKeage M. J., Galettis P., Hambley T. W., Palmer B. D., Baguley B. C. (2000). Relationships between hydrophobicity, reactivity, accumulation and peripheral nerve toxicity of a series of platinum drugs. *British Journal of Cancer*.

[3] Desoize B., Madoulet C. (2002). Particular aspects of platinum compounds used at present in cancer treatment. *Critical Reviews in Oncology/Hematology*.

[4] Extra J. M., Espie M., Calvo F., Ferme C., Mignot L., Marty M. (1990). Phase I study of oxaliplatin in patients with advanced cancer. *Cancer Chemotherapy and Pharmacology*.

[5] Gamelin E., Gamelin L., Bossi L., Quasthoff S. (2002). Clinical aspects and molecular basis of oxaliplatin neurotoxicity: current management and development of preventive measures. *Seminars in Oncology*.

[6] Lehky T. J., Leonard G. D., Wilson R. H., Grem J. L., Floeter M. K. (2004). Oxaliplatin-induced neurotoxicity: acute hyperexcitability and chronic neuropathy. *Muscle and Nerve*.

[7] Screnci D., McKeage M. J. (1999). Platinum neurotoxicity: clinical profiles, experimental models and neuroprotective approaches. *Journal of Inorganic Biochemistry*.

[8] Ling B., Coudoré F., Decalonne L., Eschalier A., Authier N. (2008). Comparative antiallodynic activity of morphine, pregabalin and lidocaine in a rat model of neuropathic pain produced by one oxaliplatin injection. *Neuropharmacology*.

[9] Ling B., Coudoré-Civiale M.-A., Balayssac D., Eschalier A., Coudoré F., Authier N. (2007). Behavioral and immunohistological assessment of painful neuropathy induced by a single oxaliplatin injection in the rat. *Toxicology*.

[10] Wolf S., Barton D., Kottschade L., Grothey A., Loprinzi C. (2008). Chemotherapy-induced peripheral neuropathy: prevention and treatment strategies. *European Journal of Cancer*.

[11] Kazuhide I., Makoto T. (2009). Microglia and neuropathic pain. *Glia*.

[12] Scholz J., Woolf C. J. (2007). The neuropathic pain triad: neurons, immune cells and glia. *Nature Neuroscience*.

[13] Miyoshi K., Obata K., Kondo T., Okamura H., Noguchi K. (2008). Interleukin-18-mediated microglia/astrocyte interaction in the spinal cord enhances neuropathic pain processing after nerve injury. *Journal of Neuroscience*.

[14] Watkins L. R., Milligan E. D., Maier S. F. (2001). Glial activation: a driving force for pathological pain. *Trends in Neurosciences*.

[15] Warwick R. A., Hanani M. (2013). The contribution of satellite glial cells to chemotherapy-induced neuropathic pain. *European Journal of Pain*.

[16] Shibata K., Sugawara T., Fujishita K., Shinozaki Y., Matsukawa T., Suzuki T., Koizumi S. (2011). The astrocyte-targeted therapy by Bushi for the neuropathic pain in mice. *PLoS ONE*.

[17] Zimmermann M. (1983). Ethical guidelines for investigations of experimental pain in conscious animals. *Pain*.

[18] Lim B.-S., Moon H. J., Li D. X. (2013). Effect of bee venom acupuncture on oxaliplatin-induced cold allodynia in rats. *Evidence-Based Complementary and Alternative Medicine*.

[19] Moon H. J., Lim B. S., Lee D. I. (2014). Effects of electroacupuncture on oxaliplatin-induced neuropathic cold hypersensitivity in rats. *The Journal of Physiological Sciences*.

[20] Kim S. K., Park J. H., Bae S. J., et al (2005). Effects of electroacupuncture on cold allodynia in a rat model of neuropathic pain: mediation by spinal adrenergic and serotonergic receptors. *Experimental Neurology*.

[21] Na H. S., Han J. S., Ko K. H., Hong S. K. (1994). A behavioral model for peripheral neuropathy produced in rat's tail by inferior caudal trunk injury. *Neuroscience Letters*.

[22] Chaplan S. R., Bach F. W., Pogrel J. W., Chung J. M., Yaksh T. L. (1994). Quantitative assessment of tactile allodynia in the rat paw. *Journal of Neuroscience Methods*.

[23] Dixon W. J. (1980). Efficient analysis of experimental observations. *Annual Review of Pharmacology and Toxicology*.

[24] Park S., Sohn S.-H., Jung K.-H., Lee K.-Y., Yeom Y. R., Kim G.-E., Jung S., Jung H., Bae H. (2014). The effects of maekmoondong-tang on cockroach extract-induced allergic asthma. *Evidence-Based Complementary and Alternative Medicine*.

[25] Gim G.-T., Lee J.-H., Park E., Sung Y.-H., Kim C.-J., Hwang W.-W., Chu J.-P., Min B.-I. (2011). Electroacupuncture attenuates mechanical and warm allodynia through suppression of spinal glial activation in a rat model of neuropathic pain. *Brain Research Bulletin*.

[26] Peters C. M., Jimenez-Andrade J. M., Kuskowski M. A., Ghilardi J. R., Mantyh P. W. (2007). An evolving cellular pathology occurs in dorsal root ganglia, peripheral nerve and spinal cord following intravenous administration of paclitaxel in the rat. *Brain Research*.

[27] Ledeboer A., Jekich B. M., Sloane E. M., Mahoney J. H., Langer S. J., Milligan E. D., Martin D., Maier S. F., Johnson K. W., Leinwand L. A., Chavez R. A., Watkins L. R. (2007). Intrathecal interleukin-10 gene therapy attenuates paclitaxel-induced mechanical allodynia and proinflammatory cytokine expression in dorsal root ganglia in rats. *Brain, Behavior, and Immunity*.

[28] Zheng F. Y., Xiao W.-H., Bennett G. J. (2011). The response of spinal microglia to chemotherapy-evoked painful peripheral neuropathies is distinct from that evoked by traumatic nerve injuries. *Neuroscience*.

[29] Di Cesare Mannelli L., Pacini A., Bonaccini L., Zanardelli M., Mello T., Ghelardini C. (2013). Morphologic features and glial activation in rat oxaliplatin-dependent neuropathic pain. *Journal of Pain*.

[30] Schröder S., Beckmann K., Franconi G. (2013). Can medical herbs stimulate regeneration or neuroprotection and treat neuropathic pain in chemotherapy-induced peripheral neuropathy?. *Evidence-Based Complementary and Alternative Medicine*.

[31] Nakanishi M., Arimitsu J., Kageyama M. (2012). Efficacy of traditional Japanese herbal medicines-keishikajutsubuto (TJ-18) and bushi-matsu (TJ-3022)-against postherpetic neuralgia aggravated by self-reported cold stimulation: a case series. *The Journal of Alternative and Complementary Medicine*.

[32] Yamada T., Kan H., Matsumoto S., Koizumi M., Sasaki J., Tani A., Yokoi K., Uchida E. (2012). Reduction in oxaliplatin-related neurotoxicity by the administration of Keishikajutsubuto(TJ-18)and powdered processed aconite root. *Gan to Kagaku Ryoho*.

[33] Gauchan P., Andoh T., Kato A., Kuraishi Y. (2009). Involvement of increased expression of transient receptor potential melastatin 8 in oxaliplatin-induced cold allodynia in mice. *Neuroscience Letters*.

[34] Nassini R., Gees M., Harrison S., De Siena G., Materazzi S., Moretto N., Failli P., Preti D., Marchetti N., Cavazzini A., Mancini F., Pedretti P., Nilius B., Patacchini R., Geppetti P. (2011). Oxaliplatin elicits mechanical and cold allodynia in rodents via TRPA1 receptor stimulation. *Pain*.

