# Supplementary material for: Gyejigachulbu-Tang Relieves Oxaliplatin-Induced Neuropathic Cold and Mechanical Hypersensitivity in Rats via the Suppression of Spinal Glial Activation
Source: Evid Based Complement Alternat Med. 2014 Nov 17;2014:436482. doi: 10.1155/2014/436482 (PMC4251814; doi:10.1155/2014/436482)
Supplement: Supplementary file 1 — Supplementary Figure 1: Three-dimentional HPLC profile of GBT. Supplementary Figure 2: Effects of GBT on oxaliplatin-induced cold hypersensitivity that was tested before GBT administration. [file 436482.f1.pdf]

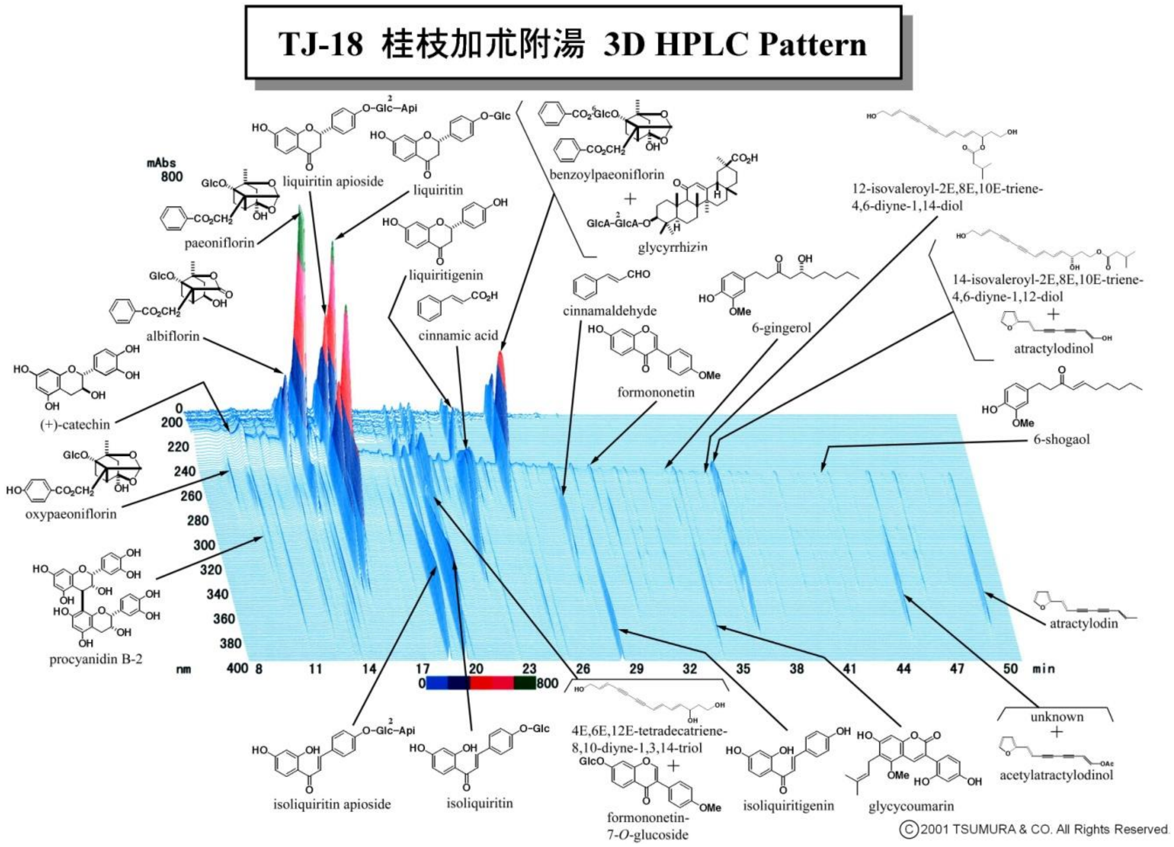

Supplementary Figure 1. Three-dimentional HPLC profile of GBT. This profile was provided by Tsumura & Co., Tokyo, Japan.

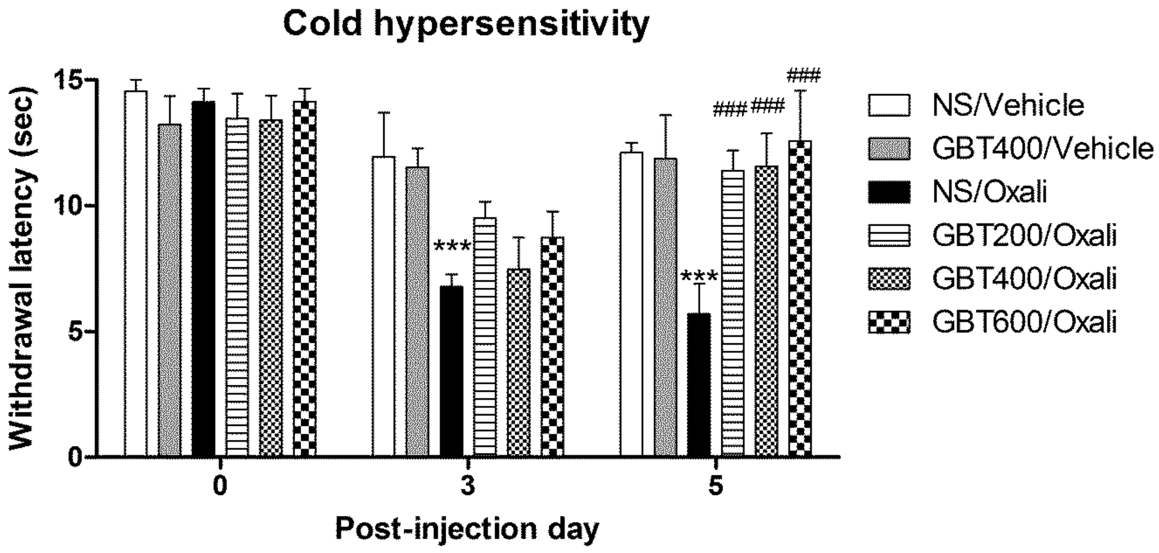

Supplementary Figure 2. Effects of GBT on oxaliplatin-induced cold hypersensitivity that was tested before GBT administration. The average tail withdrawal latency in response to cold stimuli prior to (left), and 3 days (middle) and 5 days (right) after an oxaliplatin (6 mg/kg, i.p.) injection. Data are presented as mean ± S.E.M. ****p*<0.001 vs. NS/Vehicle; ###*p*<0.001 vs. NS/Oxali by one-way ANOVA followed by Dunnett's post hoc test.
